# Physico-Mechanical and Rheological Properties of Epoxy Adhesives Modified by Microsilica and Sonication Process

**DOI:** 10.3390/ma13235310

**Published:** 2020-11-24

**Authors:** Andrzej Szewczak, Maciej Szeląg

**Affiliations:** Faculty of Civil Engineering and Architecture, Lublin University of Technology, Nadbystrzycka 40, 20-618 Lublin, Poland; a.szewczak@pollub.pl

**Keywords:** microsilica, epoxy resins, sonication, viscosity, surface free energy, strength parameters

## Abstract

Industrial waste from the production of metallic silicon and silicon–iron alloys, which includes silica fumes (microsilica), is subject to numerous applications aiming at its reuse in concrete and polymeric composites. Recycling solves the problem of their storage and adverse environmental impact. Six different formulas of epoxy resins were tested, differing in the type of polymer, the mixing process (sonication or not) and the presence of microsilica. The study showed that microsilica added to the epoxy resin changes its viscosity and free surface energy, and these are the parameters that determine the adhesion of the polymer to the concrete surface. Strength tests and SEM analysis have determined how microsilica molecules can penetrate the structure of polymer macromolecules by filling and forming temporary chemical bonds. Mixing the fillers with the adhesive was achieved by using a sonication process. The analysis of the obtained results showed that, depending on the initial composition of the polymer, the addition of microsilica can change the chemical, physical and mechanical properties of the hardened adhesive to varying degrees. In the case of adhesives used in the construction industry to strengthen and glue structural elements, these changes significantly affect the durability of the adhesive joints.

## 1. Introduction

The metallurgical processing and mining industry—as a branch of the economy supporting many other areas, including construction—is currently one of the main sources of environmental pollution. The processing of natural resources, such as metal ores and hard coal, absorbs electricity, heat and water. It is also a source of emission of a significant amount of CO_2_ to the atmosphere, sewage and other wastes to water and soil [1,2,3,4]. Despite many projects and studies aimed at improving production and directing it towards more efficient processing of supplied raw materials and limiting the emission of harmful substances, the impact of the industry on the natural environment is currently widely discussed in numerous discussion panels [5,6,7,8]. Products of raw materials processing are also a problem.

The formation of industrial wastes in the form of powders and other solid fractions, such as microsilica (MS), fly ashes, coal fumes or blast furnace slag, has caused a research trend aimed at their reuse (recycling) in various industries [9,10,11,12,13,14]. One of the ways of using this type of wastes is to use it in construction. In materials engineering, a popular trend is to search for methods to modify existing composite materials by adding recycled matter [15,16,17]. On the one hand, this process is done for the benefit of the environment; on the other hand, it helps to obtain materials with changed, improved internal structure and properties. A flagship example of this is the use of silica fumes (micro- and nanosilica) as a by-product of silicon processing and production of ferro–silicon alloys, and fly ashes (a product of fuel combustion in power plants and smelters) in cement-based composites [18,19,20,21,22,23,24].

In civil engineering, it is now becoming a common trend to change the use of existing buildings. Such projects are mainly focused on changes in the structure of the building, so that it can carry greater loads associated with the new purpose of the object. On a large scale, works are also carried out to extend the life span of buildings and other facilities, primarily utility buildings, as well as to preserve historical knowledge of past times, in the case of historic buildings. This makes it possible to extend the life span of a building without the need to construct new facilities and engage new resources [25,26]. As a result, the need to produce more building materials such as cement and steel, the production of which is one of the primary sources of CO_2_ emissions to the atmosphere, is indirectly reduced. In the case of the first group of construction works, modification of the existing structure in order to increase its load-bearing capacity and durability may be performed by using the strengthening elements, which are composite tapes with reinforcement in the form of [27,28]:

- Glass fibers—GFRP,
- Carbon fibers—CFRP,
- Aramid fibers—AFRP.

In these tapes, the appropriate fibers (glass, carbon, or aramid) are a reinforcement of the polymer matrix, made of epoxy, polyester resin and other polymers. In the case of CFRP tapes, it is possible to use MS, recycled rubber as well as carbon nanotubes for their production. Thanks to their advantageous strength to weight ratio, the reinforcing tapes allow for a significant increase in the load-bearing capacities of structural elements—most often, reinforced concrete and steel beams, columns, foundations, walls, vaults, or slab ceilings [27,28].

The glued joints are a specific type of joint. Their durability depends on the parameters of the surfaces to be bonded, mainly roughness, modulus of elasticity and surface contamination, as well as on the adhesive properties between the surfaces to be bonded. The glue (adhesive polymer) determines the effectiveness of the joint to the greatest extent. The loss of stability and effectiveness of such a bonding can be achieved by tearing off the surfaces to be bonded (loss of adhesion) or by permanent destruction of the internal structure of the adhesive (breaking of chemical bonds between polymer particles). For this reason, it is very important to carefully analyze all factors that may ultimately affect the durability of the joint [29,30]. Adhesive joints occur:

- In reinforced elements at the contact between the reinforcing tape and the element surface;
- Between materials with different mechanical properties;
- Between layers of the same material, laid out in different directions.

The development of research areas on the glued joints is currently focused on the search for new adhesives through changes in their chemical composition, and modifications of existing formulas with the use of fillers (their use allows the reduction of the amount of polymer obtained as a product of the refining and processing of crude oil). This group includes, e.g., ground limestone and dolomite, clay from ground bricks and other ceramic materials, MS, quartz powder, granite powder, basalt powder or gypsum [30,31,32]. Their dosage strictly depends on the required final properties of the obtained composite. In the case of adhesion polymers based on epoxy resins, the most important effect, which depends on the addition of a filler, is the adhesion of the adhesive to the target substrate and durability of the joint. Depending on the chemical nature of the filler molecules and on its physical parameters (shape, size, specific surface area, and density), it is possible to obtain increased adhesion of the adhesive to the target surface. It is also important that the filler addition does not reduce the value of mechanical properties, mainly modulus of elasticity, strength and, consequently, durability of the adhesive layer. This is particularly important when taking into account the possibility of aging of the polymer layer as a result of changes in its internal structure, consisting mainly in relocation of electrons and weakening of chemical bonds between molecules.

An important group of tests, described in [33,34,35,36], are analyses of flammability, thermal stability and operation of resins subjected to increased temperature and loads. The addition of a filler, the MS in this case, may significantly reduce the flammability of a composite based on an epoxy binder. This is due to the formation of structures containing SiO_2_ molecules on the surface of the composite, which prevent ignition of the resin by binding functional groups. The MS influence on the stabilization of the hardening process (the crosslinking reaction of chemosetting and thermosetting resins) is also important. The MS content must be strictly determined, because too much of it limits the effectiveness of hardening. According to [36], the presence of MS has an impact on the course of cracks arising at elevated temperatures, by changing their propagation and limiting their development.

Adhesion of the glue to the substrate depends on several factors: viscosity of an adhesive at the time of application on the surface, chemical composition of the polymer, type of filler used, roughness of the substrate, or the application method of the adhesive [37,38].

Viscosity is a parameter describing the state of cohesion and equilibrium of hydrocarbon molecules inside the polymer in liquid state, before its hardening. Its size is influenced by temperature, polymer density, types of functional groups and molecular spacing [38,39,40]. Adding a filler to the adhesive may significantly affect its viscosity and, as a consequence, the degree to which the adhesive fills irregularities occurring on the surface, e.g., concrete [39,40]. However, the problem is the correct mixing of the adhesive and the filler. This is due to the nature of the polymer and filler (polymer—liquid, filler—solid), differences in density, character and chemical composition, and reactivity of the filler. An effective method to achieve the correct degree of homogenization of the polymer and the filler is the use of ultrasonic mixing—the sonication. As the analyses [38,39,41] have shown, ultrasounds cause a number of phenomena (change of pressure and temperature of the polymer medium, activation of filler molecules as a result of the so-called sonoreaction), which lead to a homogeneous structure of the polymer with the filler in the liquid phase. The phenomena occurring during the sonication process influence the distribution of polymer and filler molecules after the resin hardening process. The sonication process itself can also be considered as a kind of modification of the glue, because the related phenomena affect the changes in the distance of chemical molecules and thus the internal structure of the polymer [42].

The surface free energy (SFE) can be used to describe the energy state and work of the adhesion at the resin-substrate contact. Its value can be determined using methods such as: the Owen–Wendt’s, Neumann’s, Fowkes’, Wu’s, Zisman’s, or van Ossa–Chauhury–Good’s. These are indirect methods, consisting in determining this energy on the basis of measurement of the angle(s) of wetting of the surface by liquids for which the dispersive-polar parameters are known, the so-called dispersive component and the polar component. Individual methods differ from each other in the type and amount of liquids used, which include, e.g., distilled water, glycerine, diodomethane, glycol or formamide [43,44,45]. The SFE is a quantity, the value of which takes into account both physical phenomena—adhesion and cohesion, or the degree of dispersion of the liquid on the tested surface, and chemical phenomena—the formation of temporary chemical bonds at the resin-substrate contact. This is particularly important in the case of adhesive polymers, whose layer is relatively thin, and the phenomena associated with the dispersive-polar state occur on its surface, in the contact layer with the substrate.

When the polymer on the target surface has already hardened, the durability of the joint over time depends largely on the mechanical parameters of the adhesive. The strength and modulus of elasticity determine the joint load-bearing capacity under the load conditions occurring, e.g., in a reinforced structure or acting on glued elements made of various materials [27,28,29,30]. The operation time of such a joint, and thus its durability is usually assumed for a period of several dozen or even several dozen years. Therefore, a possible improvement of these parameters as a result of adhesive modification is justified.

The aim of the research described in this paper is to assess the possibility of modifying the rheological, physical and mechanical properties of two types of epoxy resins (adhesive polymers) by adding inorganic filler in the form of MS. A stationary sonicator was used to mix the polymer and the filler. The research carried out allowed the determination of changes in the structure of the epoxy resin due to the MS application. The comparative analysis of the obtained results gave an answer to the question whether the MS can significantly improve the adhesive parameters determining its adhesion to the substrate (e.g., concrete, ceramic) and increase the durability of the joint after curing. The research described in the article is part of a broader research plan, concerning the possibility of the modification of epoxy resins using organic and inorganic powder fillers.

## 2. Materials and Methods

### 2.1. Components Used and Mixtures

Two types of epoxy resins, the Epidian 53 and Epidian 430 (Ciech. S.A, Nowa Sarzyna, Poland), were used in the study. The first adhesive is a pure epoxy mass, without fillers. The second one contains quartz meal with a grain size up to 0.5 mm. The polymer parameters are presented in Table 1. The exact parameters that the described resins should meet are described in [46]. Six series of samples were tested in total. For each resin, series were made according to the following scheme: unmodified resin, resin subjected to the sonication, resin with the addition of 0.5% MS (in relation to the weight of the resin) subjected to the sonication. MS (BASF Poland, Warsaw, Poland) was used, characterized by a density of 2.2 g/cm^3^, average particle size of 0.1 µm, specific surface area of 20,000 m^2^/kg, and SiO_2_ content ≥ 98%. The series and its designations are presented in Table 2.

The Z1 (Ciech. S.A, Nowa Sarzyna, Poland) amino hardener (triethletetraamine) with a density at 22 °C of 0.98 g/cm^3^ and viscosity in the range 20–30 mPa∙s was used as the hardener. The amount of the hardener was assumed according to the recommendations of the resin manufacturer in relation to the weight of the resin before hardening. The reference temperature at which the basic density and viscosity values were measured was 22 °C.

Prior to the modification, the density and viscosity of unmodified resins were established as reference values. Then the series: ER53/S, ER53/S/M, ER430/S, and ER430/S/M were subjected to a sonication process which lasted 3 min. During the sonication, the processes associated with rapid mixing of resin and MS were observed. The ultrasonic source was a stationary sonicator UP 400S (Hielscher Ultrasonics Gmbh, Teltow, Germany), emitting 24 kHz waves with power range control from 0 to 400 W, and with cycle (amplitude) control in the range 0.5–1. After the sonication the viscosity and temperature of the resins were measured again—the ultrasonic action increased the temperature of polymers.

### 2.2. Methodology

The following tests and measurements were carried out under the research program:

- Viscosity of the unmodified resin;
- Temperature and viscosity of the resin when the sonicator was switched off;
- Temperature and viscosity of the resin from the moment the sonicator was turned off until the resin reaches its initial temperature of 22 °C (reading every 5 min);
- SFE on the surface of hardened resins;
- Hardness (HV10) of the resin;
- Uniaxial tensile strength of the resin;
- Modulus of elasticity and Poisson’s number of the resin;
- Analysis of the local microstructure of the hardened resins using a scanning electron microscope (SEM).

During the cooling of the resin to 22 °C, after the sonication process, temperature and viscosity were measured every 5 min. For this purpose, a stationary rotational viscometer of the H type (FungiLab, Barcelona, Spain), an R2 spindle and a laboratory thermometer were used. Depending on the resin type, 100 rpm for ER53 and 30 rpm for ER430 was applied (the number of spindle revolutions depends on the value of resin viscosity at the initial temperature and the range of viscosity value that can be measured by the device at a given number of revolutions). The accuracy of the viscosity measurement is 0.1 mPa∙s and the temperature—0.1 °C.

For the measurement of SFE and hardness, samples in the form of 5 mm thick discs with a diameter of 100 mm were prepared. All samples were tested 14 days after the resins had hardened. SFE was determined using the Owens–Wendt method [41,43,47]. This method allows to calculate SFE on the basis of measurements of the wetting angle of the tested surface determined for two liquids with known the dispersive-polar parameters. The first liquid—distilled water—is a strongly polar liquid, its polar component of free energy is equal to 51 mJ/m^2^, while the SFE—72.8 mJ/m^2^. The value of the polar component of the second liquid—diodomethane—2.3 mJ/m^2^, while its SFE—50.8 mJ/m^2^ [41]. The SFE is the sum of the dispersive and polar components, determined according to Equations (1)–(3) [41,48]:

(1) $$\gamma_{s}=\gamma_{s}^{d}+\gamma_{s}^{p}$$

(2) $${\left(\gamma_{s}^{d}\right)}^{0.5}=\frac{\gamma_{d}\cdot \left({\cos\theta }_{d}+1\right)-\sqrt{\frac{\gamma_{d}}{\gamma_{w}}}\cdot \gamma_{w}\left({\cos\theta }_{w}+1\right)}{2\left[\sqrt{\gamma_{d}^{d}}-\sqrt{\gamma_{d}^{p}\cdot \frac{\gamma_{w}^{d}}{\gamma_{w}^{p}}}\right]}$$

(3) $${\left(\gamma_{s}^{p}\right)}^{0.5}=\frac{\gamma_{w}\cdot \left({\cos\theta }_{w}+1\right)-2\sqrt{\gamma_{s}^{d}\cdot \gamma_{w}^{d}}}{2\sqrt{\gamma_{w}^{p}}}$$

where:

γ_s_—SFE,
γ_s_^d^—dispersive component of SFE,
γ_s_^p^—polar component of SFE,
γ_d_—SFE of diodomethane,
γ_w_—SFE of distilled water,
γ_d_^d^—dispersive component of SFE of diodomethane,
γ_w_^d^—dispersive component of SFE of distilled water,
γ_d_^p^—polar component of SFE of diodomethane,
γ_w_^p^—polar component of SFE of distilled water,
θ_d_—wetting angle with diodomethane,
θ_w_—wetting angle with distille water.

The wetting angle was measured on 3 samples with the PGX goniometer (Rycobel, Deerlijk, Belgium). For each sample 10 measurements were taken. The results and photos were then entered into NIS-Elements D software (Nikon, Amsterdam, Netherlands), and SFE was calculated according to Equations (1)–(3).

**Figure 1:**
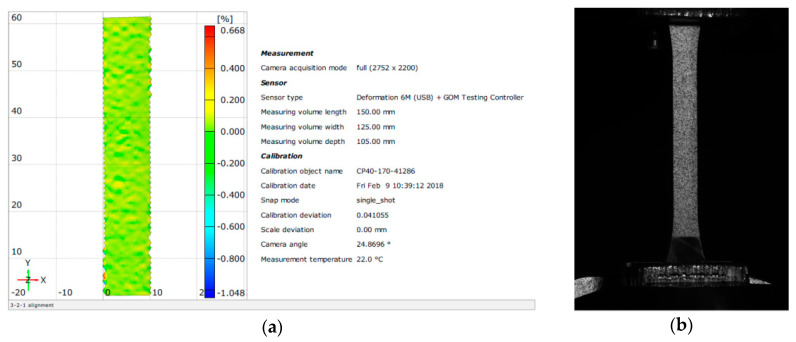
Using the ARAMIS system for the determination of mechanical parameters: (**a**) image of the specimen in the system; (**b**) specimen with the pattern applied.

The strength parameters were determined on 4 mm thick dog-bone shaped samples, according to [49,50]. Six samples were tested for each series. The uniaxial tensile strength tests were carried out using MTS 810 strength machine (MTS Systems, Eden Prairie, MI, USA) with electronic recording, and with a load head range up to 5 kN. A displacement control of 1 mm/min was adopted. The ARAMIS system (Gom a Zeiss Company, Oberkochen, Germany) connected to the strength machine was used to calculate the tensile strength and modulus of elasticity (Figure 1). This system is based on a non-contact 3D strain measurement and 2D and 3D analysis. During the loading of the element, photographs are taken using digital cameras (predefined number of photos per unit of time—500 photos/min. The system recognizes the surface of the measured object, obtained by applying an appropriate pattern on the sample, based on the contrast and location of characteristic points (each pixel in the picture is assigned appropriate coordinates). The program takes the first photo taken during the test as the zero state. Subsequent photos are taken and saved until the test is completed. Saving all pictures and entering additional output data, i.e., sample thickness and strength press data, allows the ARAMIS system to perform a thorough analysis, calculate displacement, strain and other strength parameters. All tests were carried out 14 days after the samples were formed and the resins hardened.

The surface hardness (3 samples per series) was measured using the Vickers’ method. The Vickers’ multifunctional hardness tester (ZwickRoell GmbH, Ulm, Germany) with a load range of 0–200 N was used. The base load was 10 N (HV10 hardness). The measurements were taken and automatically read out on the basis of the imprint formed after the pyramid was pressed into the resin surface. The hardness was measured as the ratio of the force applied on the sample surface to the imprint surface. For each sample, 10 hardness measurements were taken.

The analysis of a local microstructure morphology in the micro-area was determined with the scanning electron microscope (SEM)—Quanta 250 FEG (FEI, Hillsboro, OR, USA). The microscope was equipped with a chemical composition analyzer based on an X-ray energy dispersion (EDS) and an electron gun with LaB6 cathode. The analysis was performed in high vacuum mode. The samples were fixed with carbon tape to aluminum holders before testing. A carbon electrically conductive layer with a thickness of about 50 nm was obtained by a sputtering process in the Quorum Q150T (Quorumtech, Laughton, UK). The test was carried out in the secondary electron spectrum, with acceleration voltage in the range 10^−15^ keV.

## 3. Results and Discussion

### 3.1. Physical Properties

#### 3.1.1. Viscosity under Initial Conditions

The starting temperature at the time of potential resin application was applied as 22 °C. The viscosity of the resins at this temperature for the individual series was equal to:

- ER53—1.488 Pa·s,
- ER53/S—1.440 Pa·s,
- ER53/S/M—1.155 Pa·s,
- ER430—197.067 Pa·s,
- ER430/S—193.333 Pa·s,
- ER430/S/M—263.733 Pa·s.

The application of the sonication process only resulted in a decrease in the initial viscosity of both epoxy resins. The ER53/S and ER430/S were characterized by viscosity lower by 3.2% and 1.9% respectively in comparison to unmodified ER53 and ER430 resins. The MS modification resulted in a differentiated effect. In case of the Epidian 53, the viscosity value was 22.4% lower. In case of the Epidian 430, the addition of filler increased viscosity by 33.8%.

#### 3.1.2. Viscosity Change as a Function of Time and Temperature

**Figure 2:**
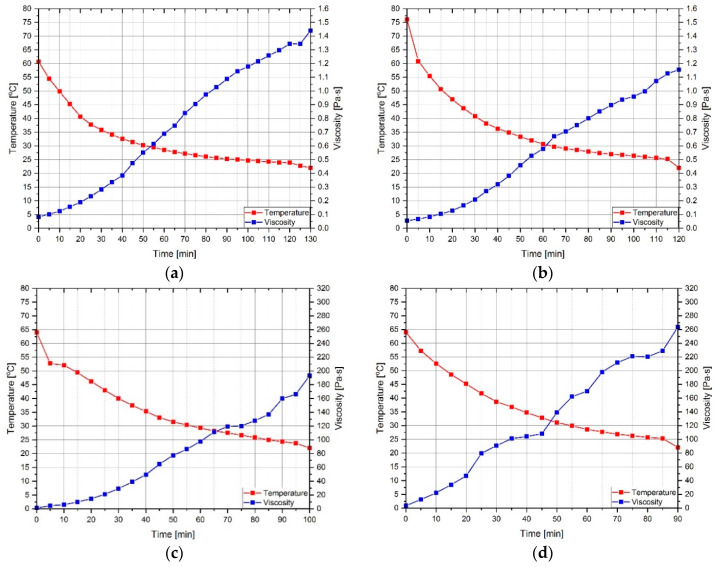
Viscosity and temperature of resins as a function of time, from the moment the sonicator was turned off: (**a**) ER53/S; (**b**) ER53/S/M; (**c**) ER430/S; (**d**) ER430/S/M.

**Figure 3:**
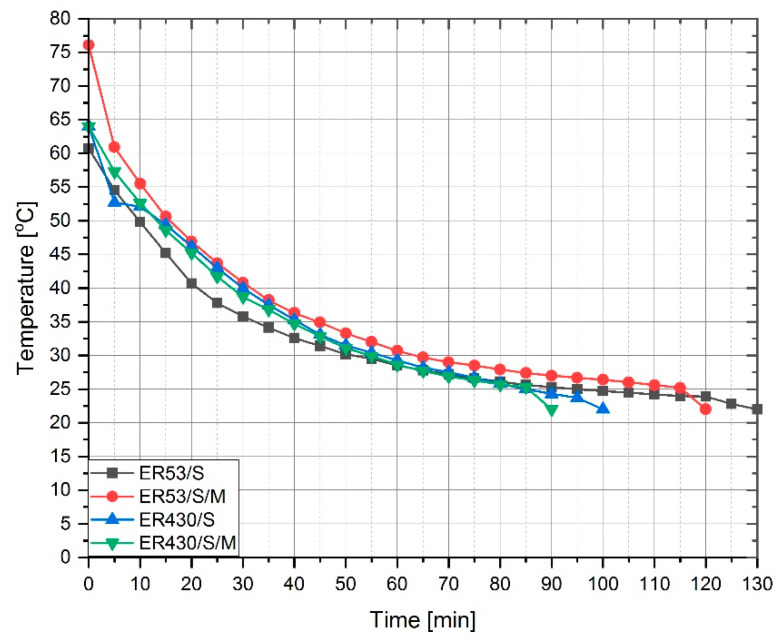
Temperature drop of individual resin series after the sonication process.

The change of viscosity and temperature of individual series of modified resins is shown in Figure 2, while Figure 3 shows a collective decrease in resin temperature over time to the initial temperature—22 °C.

To explain the relationships presented, reference should be made directly to the chemical composition of resins and the processes caused by sonication, which are described in detail in [38,39,40,41]. In atomic terms, resins are organic compounds of carbon, oxygen and hydrogen. Their appropriate configuration, including the occurrence of epoxy groups and sequences (mers) that are repeated in the polymer structure, undergoes initial reorganization under the influence of ultrasounds. In the studies described in [51], it was noted that the power of the applied ultrasounds, the speed of their propagation in the medium and the amplitude are of great importance. Larger conglomerates are broken up as a result of vibrations, the temperature of the medium increases and, locally, the pressure of the medium changes. It causes a decrease in viscosity, which was also shown and confirmed in [52,53]. An additional phenomenon, described in [41,42,54,55], caused by the ultrasonic cavitation (a phenomenon responsible for thermodynamic changes in the volume of the resin) is the formation of micro-bubbles containing resin fumes. Their rapid formation and collapse due to pressure changes additionally cause a decrease in viscosity during sonication. It is worth noting that for the ER53/S/M the temperature to which the resin was heated is higher than for the ER53/S. This may be due to a partial heat accumulation by the MS particles. At the same time, the time when the resin has reached the initial temperature of 22 °C is about 10 min shorter, which indicates faster heat release during cooling. The reduction in viscosity is relatively small. It is an effect of secondary ordering of the structure of mers and functional groups in the volume of the resin. When returning to the state of physical equilibrium, particles form a more orderly and compact structure. The MS particles, whose molecules can form temporary bonds, i.e., hydrogen bonds with polymer molecules, as also shown in [56], and interacting by the Van der Waals and London forces, results in a further decrease in viscosity. This is an effect of sharing electrons from the electron cloud excited during sonication from the polymer and MS molecules (silica contained in MS has the same number of valence electrons as carbon—4). So it is possible to temporarily bond silica molecules to mers, with simultaneous permanent or temporary relocation of oxygen and hydrogen atoms. As a result, the structure is further thickened (silica has a higher molar mass than carbon), and viscosity is reduced. It is also possible to partially merge the free ends of the polymer chain elements.

With the ER430 series, similar phenomena occur between MS and resin. The temperature reached by the samples at the end of sonication is similar, due to the heat accumulation not by the MS particles but by the quartz powder contained in the resin. As in the case of ER53, the sample with the addition of microsilica returned to its initial temperature faster. At the same time, due to the generally faster heat transfer from quartz powder, the time to return to this temperature is on average about 30 min shorter for the ER430. A similar degree of viscosity reduction was also found between the ER430 and ER430/S series. The exception is the ER430/S/M, which should be explained by the fact that silica derived from quartz powder can partially combine with particles of the polymer chain via MS particles. The structures created in this way are possible due to the reactive nature of MS, which was additionally excited in the sonication process. It is also possible that the SiO_2_ molecules contained in the quartz powder achieve partial reactivity. As a result, the use of Epidian 430 for, e.g., gluing materials with different properties, or gluing reinforcing tapes on the surface of concrete or steel can be more effective—the adhesive with lower viscosity can better fill in irregularities in the substrate, in case of a strongly rough surface. On smooth or sanded surfaces, on the other hand, the higher viscosity adhesive can bond faster and more effectively.

The analysis of the results showed that the viscosity of the modified adhesive before hardening directly affects the initial adhesion of the adhesive to the substrate. This is also confirmed by the conclusions contained in [54,56]. The use of sonication at the time of bonding results in more durable bonds between the substrate and a reinforcing tape, mainly due to the favourable changes in viscosity and surface tension of the resin.

#### 3.1.3. Surface Free Energy—SFE

**Figure 4:**
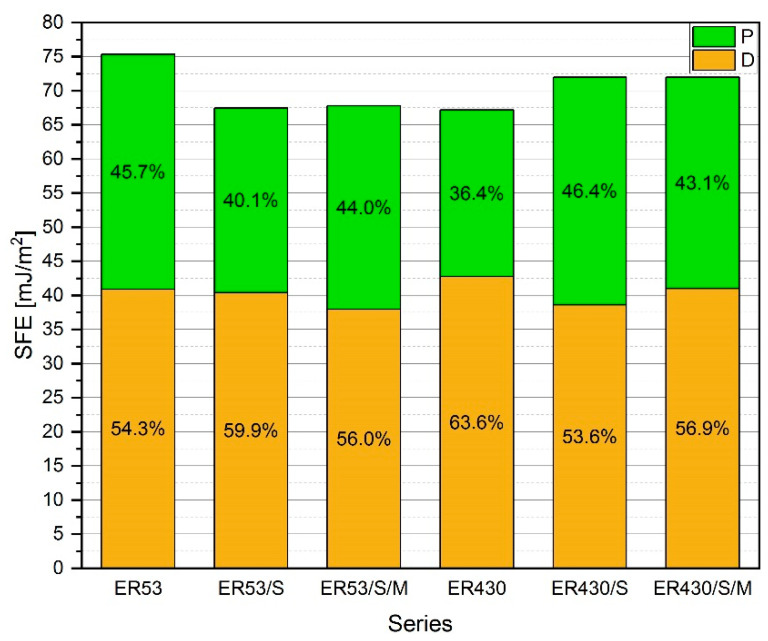
Surface free energy (SFE) for individual resin series, including the percentage of dispersive component (D) and polar component (P).

In Figure 4 and Table 3 the results of the SFE and the individual components, i.e., dispersive and polar, are shown. The obtained results differ depending on the type of resin and the modification applied. In the case of Epidian 53, the highest SFE value was reached by the unmodified resin, i.e., ER53. The use of sonication (ER53/S) and modification of the resin with MS (ER53/S/M) resulted in a very similar effect, i.e., a decrease in SFE value by 10.4% and 10.0% respectively. In the case of Epidian 430, the effect resulting from the modification was the opposite—an increase in SFE by 7.1% and 7.4%, respectively, for ER430/S and ER430/S/M, compared to unmodified resin—ER430. For all series, the share of the dispersive component in the total SFE value was higher, ranging between 53.6–63.6%. The polar component ranged between 36.4–46.4% of the SFE value. The value of the dispersive component, depending on the series, was within a fairly narrow range of 37.97–42.73 [mJ/m^2^]. The applied modification, however, had a greater impact on the polar component, as the obtained values for individual series were within a wider range—24.43–34.43 [mJ/m^2^]. The value of the coefficient of variation only in one case slightly exceeds 5%, and in the vast majority of cases it is in the range of 1–3%.

The analysis of the SFE results, in relation to the tested material, which are adhesives, made it possible to determine the influence of the applied modifications on the final adhesion (after hardening reaction) to the target application surface. The chemical composition and the effect of ultrasounds have a great influence on the final values of the individual components, similarly to viscosity [51,52]. In the case of ER53, the decrease in SFE values resulted from a decrease in the mainly polar component, which depends largely on intermolecular interactions. As mentioned earlier, these interactions take place mainly inside the resin structure, which after curing is characterized by lower polarity and adhesion of the external surface resulting from chemical interactions. These phenomena are also partially explained in [56], by describing the process of partial dislocation of free joints in polymer chains by connecting them by means of hydrogen bonds with SiO_2_ particles. It should be noted, however, that the dispersive components, depending on physical phenomena on the boundary phase of the solid (cured resin)—measuring liquid, i.e., the degree of dispersion, change to a much smaller extent. This is directly related to the results of viscosity measurements—as noted earlier, lower viscosity allows for easier dispersion of the resin in liquid state on the target surface during bonding. The slight decrease in the dispersive value and the increase in the polar component of the ER53/S/M compared to the ER53/S is due to the presence of MS and its reactive particles. The MS, as confirmed by the results of the tests described below, concentrates at the resin surface after hardening. Nevertheless, the resin retains its adhesive parameters.

The series made of Epidian 430 showed other relationships. As in the case of viscosity tests, the results were largely determined by the presence of quartz powder and the phenomena associated with the effects of ultrasounds and the MS addition. Both modified series recorded an increase in SFE, thus improving the final adhesion of the adhesive to the substrate. However, unlike the ER53, this increase was caused by a change in the polar component. Adsorption of the polymer in the liquid state to the surface of the application will be more due to the polar interaction at phase contact. The excitation of SiO_2_ molecules, which are composed of quartz and MS, causes a certain orientation of dipoles and charges on the surface of the hardened resin, which is characterized by a vitreous structure in the form of a solid. The studies presented in [57] show that the use of micro- and nano-silica may increase the degree of crystallinity of the composite. During the crosslinking reaction polar interactions and bonding between the resin and the substrate occur, mainly resulting from the exchange or sharing of electrons and the formation of chemical bonds, also dependent on the chemical nature of the substrate itself. The MS addition binds some of the molecules derived from quartz powder, which causes a slight decrease in the polar component of SFE. It is worth noting, however, that the dispersive component remains constant, so it is possible to maintain appropriate adhesion resulting from the ability of the resin in the liquid state to form mechanical bonds (interlocking of the resin with uneven substrate). It is also related to the phenomena described in [58,59] at the contacts of two phases. Adhesion of the resin (liquid phase) before curing depends primarily on the degree of its dispersion and mechanical adhesion, viscosity and surface tension. During and after the hardening, at the solid–solid boundary further adhesion depends on the interactions between the surface layers of the combined materials. The research described in [54,56] also shows that it is possible not only to activate the resin in the liquid state by ultrasounds, but also to interact with it, e.g., on a reinforcing tape, as its structure allows the ultrasounds to propagate and partially activate adhesion on the tape surface. The degree of wettability of the surface, e.g., of the tape or the materials to be bonded by the resin, is also increased. The described processes, however, do not disturb the generally accepted molecular and thermodynamic description of phenomena at the phase contact [59], which could cause, e.g., a sudden rupture of the glued connection. The SFE components remain in relatively constant relation to each other.

### 3.2. Mechanical Properties

#### 3.2.1. Hardness—HV10

**Figure 5:**
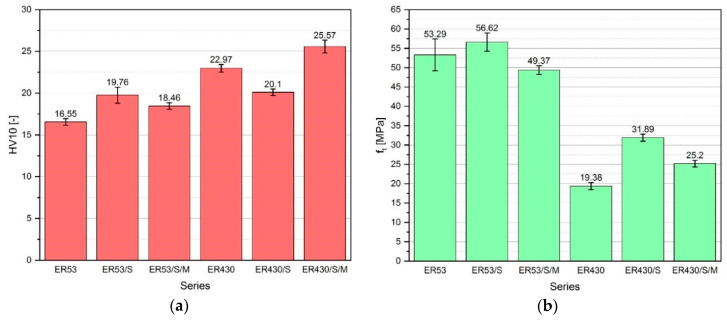
Results of the mechanical properties of individual resin series: (**a**) the HV10 surface hardness; (**b**) the uniaxial tensile strength; error bars indicate the standard deviation.

The values of the HV10 surface hardness of resins, depending on the modification applied, are shown in Figure 5a. Resin with quartz powder and its modifications (ER430, ER430/S, and ER430/S/M) were characterized by higher hardness than resins based on Epidian 53, on average by 25.3%. The sonication process itself caused a different effect depending on the type of resin. In case of ER53/S the hardness value was 19.4% higher than ER53. However, the relationship between ER430/S and ER430 was the opposite of the above, and the obtained difference between the series was 12.5%. Modification of the resin in the form of sonication with simultaneous MS application resulted in an increase in its hardness, obtaining for the ER53/S/M and ER430/S/M values higher by 11.5% and 11.3% respectively from the ER53 and ER430.

The HV10 results confirm the analyses carried out during the viscosity and SFE tests and are a prelude to the analysis of mechanical properties, which were carried out at the end of the established test program. It should be noted that hardness is the value tested on the surface of the hardened resin. In adhesive polymers, the properties of the joint are determined by an upper, thin adhesive layer, directly in contact with the surfaces to be bonded. The paper [60] describes how the way the adhesive is applied, processed, and the thickness of the adhesive layer, affects the bond between the CFRP tape and a steel beam surface. Therefore, the hardness can be considered as an indirect measure of the durability of a thin polymer layer that occurs in bonded joints. In the results obtained, it was noted that in the Epidian 53 resin series, the modifications applied, due to the arrangement of the internal resin structure by sonication, resulted in a more effective hardening of the resin. The difference between the ER53/S and ER53/S/M series results from the formation on the resin surface a layer composed of MS and fragments of polymer chains, mainly on the basis of chemical interactions described in [54]. This layer is characterized by lower hardness and higher elasticity compared to the surface of the resin subjected only to the sonication. In [61], the results of hardness tests of MS modified epoxy resin are presented. It was shown that its addition may increase the value of hardness, but to some extent its content—up to 2%. Further increase of the MS content causes a slow decrease in hardness. The best result was obtained by combining the addition of MS and carbon fibers.

The presence of quartz powder in resins based on Epidian 430 changed the observed relationships. The reduction of hardness for the ER430/S may result from the shift of quartz powder grains to the center of the sample and the formation of the resin layer on its surface. In the ER430/S/M, MS together with the quartz powder forms a more compact structure on the surface (MS particles combine with quartz powder to stabilize its layer on the sample surface). The effect obtained in this way may have a positive impact on the durability of the modified resin coating on its surface. In the studies described, it can be observed, additionally from also [61], that quartz powder, which is characterized by absolute hardness equal to 100 according to the Mohs’ scale, in combination with MS, creates a similar effect as in the case of a combination of MS and dispersed reinforcement due to the dynamic sonication processes.

#### 3.2.2. Uniaxial Tensile Strength

Figure 5b shows the results of the tensile strength (f_t_) of resins depending on their modification. Definitely higher f_t_ values were obtained by samples made of the Epidian 53. They were on average 108.3% higher than resins made of the Epidian 430. The sonication process resulted in a very positive effect on the increase in resin strength, which is particularly visible for the Epidian 430. The f_t_ value for ER53/S was 6.2% higher than ER53, and for ER430/S the f_t_ value was 64.6% higher than for ER430, which also confirms the results obtained in [56,58,62]. The obtained effect of MS modification differed depending on the type of resin. Thus, in the case of ER53/S/M the f_t_ value was 7.4% lower compared to ER53. However, in case of the series with the Epidian 430, ER430/S/M had a higher f_t_ value than ER430 by 30.0%.

The tensile strength is one of the basic parameters (apart from the shear and tear strength) determining the durability and reliability of a glued joint [60,63]. In the case of testing the modified resin itself, the f_t_ value depends largely on the degree of packaging of the polymer molecules in its structure, and the chemical bonds formed during sonication and crosslinking reaction and their durability, as well as the presence of fillers—factors which thus influence the previously described parameters. During the test, the sample rupture occurs suddenly, the value of relative elongation and deformation in the direction of tensile force is low [64,65]. The rupture of the structure results from the cracking of chemical bonds, and thus, the relocation of some molecules as a result of the disturbance of their energy state, determined during hardening, associated, among others, with the Grifitth’s theory [64]. According to this theory, the fracture of a brittle polymer occurs as a result of damage occurring at specific points, most often discontinuities of the material caused by inclusions in the form of larger groups of molecules or the presence of atoms from fillers. Changes in the elastic energy, which is concentrated around these microcracks, finally create a new surface during sample rupture. Such cracks may be the defects described in [66], running perpendicularly to the direction of polymer flow during its formation. It is therefore important to analyze the molecular structure obtained by adding a filler.

The ER53/S and ER430/S series are characterized by the highest degree of particle packing, mainly caused by the sonication. When analyzing the ER53/S/M, it should be noted that MS can weaken this structure to some extent, in relation to the hardened resin, which is also confirmed by the results presented in [57]. By comparing these data with the hardness results, it confirms the conclusion that in the case of ER53, MS accumulates mainly on the surface of the sample, forming a thin layer of SiO_2_, partially bonded to the polymer chains. As a result, there are fewer of these bonds inside the material structure.

In the ER430, the previously described shifting of silica from the quartz powder to the interior of the resin during hardening and the phenomena also known from [56], strengthens this structure. The result is a significant increase in the tensile strength for the ER/430/S. At the same time, the material is weaker in the surface layer, which may also be associated with the wall effect (at the interface between the polymer and the mold more resin accumulates). In the ER430/S/M this effect is centered. Although the material is characterized by a lower f_t_ value, but MS and quartz powder, excited energetically during sonication, form a different structure. It is more homogeneous at the sample surface and slightly weaker inside the sample, but it is nevertheless more compact due to the sonication.

#### 3.2.3. Young’s Module and Poisson’s Number

**Figure 6:**
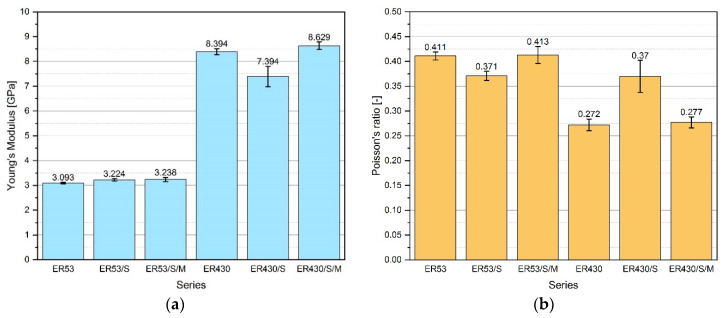
Values of mechanical properties of modified epoxy resins: (**a**) Young’s module; (**b)** Poisson’s number; error bars indicate standard deviation.

The values of Young’s module and Poisson’s number of individual resin series are shown in Figure 6. The Young’s module for resins made of Epidian 53 reached significantly lower values in comparison to series based on Epidian 430. The difference was as much as 60.9%. The method of resin modification did not significantly affect the value of Young’s module for resins based on Epidian 53, because ER53/S and ER53/S/M obtained values higher than ER53, respectively, by 4.2% and 4.7%. It results directly from the already described dependencies and the degree of ordering the internal structure of resins. The effect of the applied modifications is more visible for resins with the quartz powder, especially when only sonication process was applied, i.e., the Young’s module value for ER430/S is lower by 11.9%. In the case of resin modification with MS and sonication the opposite effect was obtained—for ER430/S/M the Young’s modulus value is 2.8% higher compared to ER430. In this case too, the conclusions of the tensile strength analysis are confirmed.

Resins made of Epidian 53 were characterized by an average 30.0% higher the Poisson’s number than resins based on Epidian 430. In the case of both resins, a very similar effect was obtained in the case of structure modification with the simultaneous use of MS and sonication, i.e., a slight increase in the Poisson’s number value—0.5% and 1.8% for ER53/S/M and ER430/S/M, respectively, compared to ER53 and ER430. This difference is within the limits of standard deviation, also may be statistically insignificant. Modification with sonication only resulted in a different effect depending on the type of resin. For ER53/S, the Poisson’s number was 9.7% lower than ER53. The same relationship, but between ER430/S and ER430 assumed the opposite relationship, with a difference in value of 36.0%.

The obtained composites and their parameters obtained in the uniaxial tensile strength testing allow to supplement the analysis of mechanical properties. The materials tested should be classified as anisotropic, brittle, characterized by relatively small deformation in the longitudinal and transverse direction. This is most evident in the ER430/S series, where a decrease in modulus of elasticity caused a significant increase in the Poisson’s number. The theoretical issues presented in [64,66] are also confirmed here.

### 3.3. Analysis of a Local Microstructure

**Figure 7:**
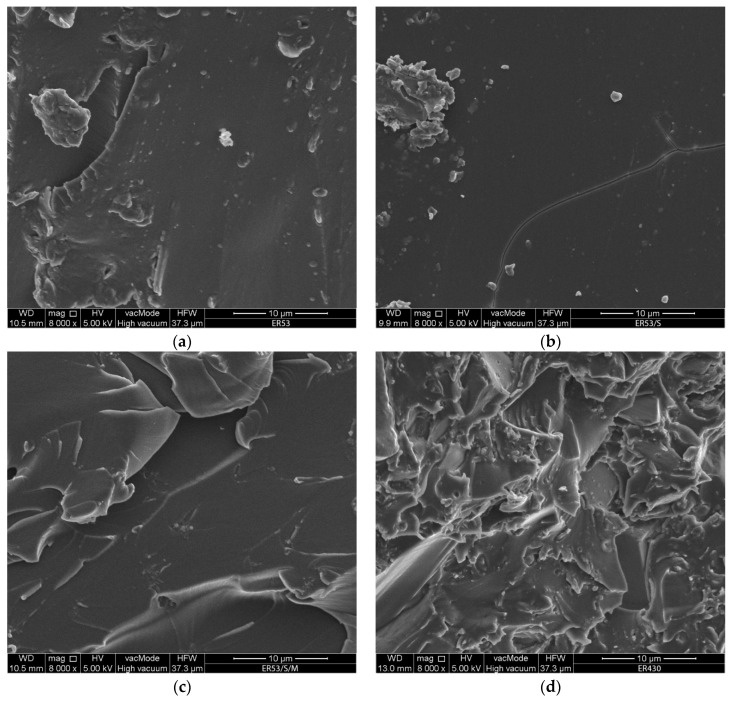

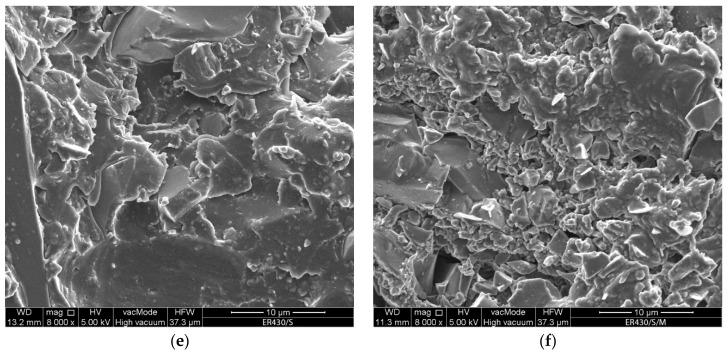
Morphology of the fracture surface of modified epoxy resins: (**a**) ER53; (**b**) ER53/S; (**c**) ER53/S/M; (**d**) ER430; (**e**) ER430/S; (**f**) ER430/S/M.

The analysis of the surface morphology of the epoxy resin fractures, using SEM images (Figure 7), finally confirmed the observations made. Unmodified resins are characterized by a less continuous structure, caused by lower packing of molecules in the polymer chains (Figure 7a,d). Arrangement of the ER53/S resin structure (Figure 7b) has a positive effect on increasing f_t_ and HV10. In ER53/S/M it is easy to notice a less homogeneous structure (Figure 7c), which results from the presence of structures connected by interactions in the form of hydrogen bonds, as described in the analysis of viscosity results. MS particles bond part of the free polymer chains formed during sonication. At the same time, the internal structure before hardening is characterized by a lower viscosity. This allows liquid resins to fill the unevenness of the substrate more effectively and to create a mechanical adhesion. The polar component, which value is higher than for the ER53/S, results from the presence of MS particles at the ends of the chains.

In the ER430, however, lumpy structures containing silica derived from both filler and the quartz powder were noticed. This confirms the earlier conclusion that it is possible to activate a part of SiO_2_ of the quartz powder by ultrasounds. The sonication (ER430/S) arranged the structure (Figure 7e), which translates into a higher f_t_ value, while making the material more brittle. The ER430/S/M (Figure 7f) is the most diverse in terms of internal structure. The MS addition resulted in additional microstructures, which are also associated with the fact that the MS particles are concentrated closer to the surface of the sample, forming a more durable coating there. The result is a higher SFE polar component, tensile strength, modulus of elasticity and hardness than for unmodified resin.

### 3.4. Correlation Analysis

Table 4 presents a correlation matrix taking into account the physical and mechanical parameters of modified epoxy resins. Two correlation coefficients were taken into account in the correlation analysis, i.e., the Pearson’s (r) and Spearman’s (ρ). The application of these two coefficients allows the determination of the strength and type of correlation between the variables. The Pearson’s correlation coefficient determines the strength of linear correlation, while the Spearman’s correlation coefficient determines the strength of any monotonous correlation, which does not have to be linear. The interpretation of both coefficients is the same, while comparing the values of both coefficients allows the determination of whether the occurring relation is more linear or non-linear.

The global correlation analysis indicates a more frequent and stronger occurrence of linear than non-linear relationships. It was noted that the resin viscosity is practically completely correlated with f_t_ and Young’s modulus (|r| = 0.94 and 0.99) and very highly correlated with HV10 and Poisson’s number (|r| = 0.86 and 0.84). This indicates that the state of the resin in the liquid phase, and thus its ability to apply correctly on the glued surface, has a very large impact on its properties in the hardened state. However, no strong correlation between viscosity, SFE and its components was found, i.e., the state of the resin in the liquid phase does not significantly affect the final adhesion of the resin to the substrate. The remaining resin parameters after hardening—HV10, f_t_, Young’s modulus and Poisson’s number—are usually very strongly or completely correlated with each other. The Young’s modulus is characterized by practically complete inverse correlation with f_t_ (r = 0.98). A similar relationship exists between the number of Poisson and HV10. The occurrence of both these relationships is characteristic of hardened epoxy resin. Additionally, between Young’s module and HV10 there is practically a full correlation (|ρ| = 0.94), but it indicates a more non-linear than linear relationship. The properties of the hardened resin, on the other hand, do not have much influence on the final adhesion of the resin to the bonded substrate, because the correlations between SFE and its components with the remaining parameters are usually weak or low. The above analysis also indicates the possibility of effective estimation of individual resin characteristics, especially in the area of viscosity, hardness and mechanical properties of the hardened resin.

## 4. Conclusions

The paper presents the results of research on the physico-mechanical and rheological properties of epoxy resins modified by sonication process and addition of filler in the form of MS. An analysis of the surface morphology of the materials tested was also performed. On the basis of the literature review, the research carried out and the analysed results and their interpretation, final conclusions were formulated:

- The analysis of changes in the viscosity of the resins allowed the assessment of the potential initial resin adhesion to the substrate. Modification of the material with the use of sonication resulted in the formation of more durable bonds at the contact between the resin and the substrate, due to a favorable change in viscosity. However, the MS effect depends on the properties of the resin itself. In the case of pure Epidian 53 resin, the filler caused a further favorable reduction in viscosity. On the other hand, additional densifying of the Epidian 430 structure with quartz powder resulted in an increase in its viscosity.
- The SFE analysis made it possible to assess potential final adhesion (after hardening) at the resin-substrate contact. The applied modifications resulted in a decrease of SFE value in the case of Epidian 53 by 10–10.4%. However, in the case of Epidian 430 the SFE value increased by 7.1–7.4%. The main factor causing the changes in this property is the sonication process. The MS influence was marginal, mainly related to the increase in the polar component. In the case of all tested series, the mechanical adhesion at the contact between resin and substrate is mainly responsible for the final adhesion—the share of the dispersive component is between 53.6–63.6% of SFE.
- A higher surface hardness was obtained in the case of resins based on Epidian 430. While the MS modification resulted in an increase in HV10 value for both resins, the sonication process itself resulted in a different effect. The ER53/S was characterized by a higher surface hardness than ER53 due to the arrangement of the polymer chains structure. In the case of ER430/S, on the other hand, there was a decrease in HV10 value, which was caused by the shift of quartz powder particles to the center of the sample and the remaining elastic resin layer at the surface, due to the influence of ultrasounds.
- The series based on Epidian 53 had on average twice the tensile strength in comparison with resins made of Epidian 430. The direct cause of such dependence is the presence of quartz powder in Epidian 430, which causes the formation of discontinuities in the structure. Arrangement of the structure by means of sonication resulted in an increase in f_t_ values, especially of ER430/S series (f_t_ increase by 64.6%).
- Correlation analysis showed, first of all, strong relationships between mechanical properties, and between viscosity and mechanical properties of resins.

## Figures and Tables

**Table 1:** The resins used in the research and their basic parameters.

Resin	Epidian 53	Epidian 430
Form	yellow, dense liquid	grey mass with addition of fine quartz sand fraction up to 0.5 mm
Flashpoint (°C)	75	170
Gelation time (min)	60	120
Epoxy number (mol/100 g)	0.4	-
Density (22 °C) (g/cm^3^)	1.12–1.15	2.04
Viscosity (22 °C) (Pa·s)	0.9–1.5	200
Solubility	ketones, esters, alcohols	

**Table 2:** Series and its designations.

Series Designation	Type of Resin	Type of Additive/Modification	Amount of the Filler (%)	Amount of the Hardener (%)
ER53	epoxy	-	-	10
ER53/S		sonication	-	10
ER53/S/M		sonication + MS	0.5	10
ER430	epoxy with mineral filler	-	-	3
ER430/S		sonication	-	3
ER430/S/M		sonication + MS	0.5	3

**Table 3:** Values of the dispersive (D) and polar (P) components of SFE with coefficients of variation.

Series	SFE (mJ/m^2^)	D (mJ/m^2^)	P (mJ/m^2^)	Coefficient of Variation (%)		
				SFE	D	P
ER53	75.33	40.90	34.43	1.7	2.4	1.3
ER53/S	67.47	40.40	27.07	1.3	2.2	3.1
ER53/S/M	67.77	37.97	29.80	1.0	4.0	2.7
ER430	67.17	42.73	24.43	1.4	3.3	3.1
ER430/S	71.97	38.60	33.37	1.6	4.8	3.6
ER430/S/M	72.00	41.00	31.00	2.0	1.0	5.9

**Table 4:** Pearson’s and Spearman’s correlation coefficient matrix.

		Pearson’s Correlations (r)							
		HV10	f_t_	Young’s Modulus	Poisson’s Ratio	SFE	D	P	Viscosity
**Spearman’s correlations (ρ)**	**HV10**	−	−0.81	0.86 *	−0.94 *	−0.23	0.45	−0.41	0.86 *
	**f_t_**	−0.77	−	−0.98 *	0.86 *	0.08	−0.43	0.27	−0.94 *
	**Young’s Modulus**	0.94 *	−0.89 *	−	−0.87 *	−0.01	0.40	−0.20	0.99 *
	**Poisson’s number**	−0.89 *	0.77	−0.77	−	0.23	−0.70	0.52	−0.84 *
	**SFE**	−0.26	0.20	−0.20	0.31	−	−0.02	0.89 *	0.08
	**D**	0.54	−0.54	0.43	−0.77	−0.09	−	−0.48	0.34
	**P**	−0.37	0.26	−0.31	0.37	0.94 *	−0.26	−	−0.08
	**Viscosity**	0.83 *	−0.77	0.77	−0.89 *	0.14	0.77	0.03	−

* correlation is significant at the 0.05 level. Designation of correlation strength: |*r*| < 0.2—weak correlation; |*r*| ∈ 〈0.2 ÷ 0.4)—low correlation; |*r*| ∈ 〈0.4 ÷ 0.6)—moderate correaltion; |*r*| ∈ 〈0.6 ÷ 0.8)—high correlation; |*r*| ∈ 〈0.8 ÷ 0.9)—very high correlation; |*r*| ∈ 〈0.9 − 1.0〉—correlation virtually complete.
